# The Impact of Clonal Hierarchy and Heterogeneity on Phenotypic Manifestations of Myelodysplastic Neoplasms

**DOI:** 10.3390/cancers14225690

**Published:** 2022-11-19

**Authors:** Siba El Hussein, Sanam Loghavi

**Affiliations:** 1 Department of Pathology, University of Rochester Medical Center, Rochester, NY 14607, USA; 2 Department of Hematopathology, The University of Texas MD Anderson Cancer Center, Houston, TX 77030, USA

**Keywords:** myelodysplastic syndrome, myelodysplastic neoplasm, MDS, clonal heterogeneity, clonal succession, molecular, scoring system, flow cytometry analysis

## Abstract

**Simple Summary:**

In this review, we provide a brief account of recent genomic findings, updated guidelines and emerging promising tools to help refine our diagnostic abilities in the setting of myelodysplastic neoplasms; We also discuss limitations of current practices during disease follow-up, to shed light on the pressing need to enhance our prognostic armamentarium in the near future.

**Abstract:**

Until recently, conventional prognostication of myelodysplastic neoplasms (MDS) was performed using the revised International Prognostic Scoring System (IPSS-R), with additional adverse prognoses conferred by select mutations. Nonetheless, the clonal diversity and dynamics of coexisting mutations have been shown to alter the prognosis and treatment response in patients with MDS. Often in the process of clonal evolution, various initial hits are preferentially followed by a specific spectrum of secondary alterations, shaping the phenotypic and biologic features of MDS. Our ability to recapitulate the clonal ontology of MDS is a necessary step toward personalized therapy and the conceptualization of a better classification system, which ideally would take into consideration all genomic aberrations and their inferred clonal architecture in individual cases. In this review, we summarize our current understanding of the molecular landscape of MDS and the role of mutational combinations, clonal burden, and clonal hierarchy in defining the clinical fate of the disease.

## 1. Introduction

Myelodysplastic syndromes (MDS) are now collectively referred to as myelodysplastic neoplasms by the WHO 5th edition, with the abbreviation “MDS” preserved [1] to emphasize the definition of MDS as clonal hematopoietic stem cell neoplasms and harmonize the terminology with myeloproliferative neoplasms (MPN). The pathogenesis of MDS includes initial ancestral lesions, often in the form of early CHIP (clonal hematopoiesis with indeterminate potential)-type or CHOP (clonal hematopoiesis with oncogenic potential)-type alterations followed by the acquisition of additional genetic aberrations (late and/or transforming events) that result in MDS, often with a highly diverse clonal hierarchy. The clonal sweeping of existing subclones by driver mutations play a critical role in MDS disease progression, refuting the previously accepted theory of linear evolution [2,3]. Deciphering mutational clonal trajectories uncovers key events that drive leukemic evolution in myeloid neoplasms. In fact, mutational hierarchical configuration can potentially delineate the fate of myeloid neoplasms and further allow us to predict the prognosis and clinical course of a disease.

Deciphering the clonal evolution of MDS up to its cell of origin is not feasible in most cases in the clinical setting; however, several investigations have provided valuable insights into the role of driver mutations in determining the trajectory of MDS development. Clonal hierarchies are best characterized using single-cell sequencing methods, preferably on longitudinal samples. A precise reconstruction of clonal ontology could be achieved by cross-sectional analysis of clonal architecture through single-cell sequencing [4,5]. Categorizing genetic aberrations into dominant versus secondary classes may enhance our understanding of MDS pathogenesis, as different driver mutations have distinct effects on disease progression and phenotypic manifestations in MDS as shown in multiple studies that combined large genotyping data sets [3,6,7,8], whole exome sequencing (WES), and targeted sequencing data [9].

Until recently, conventional prognostication of MDS was performed using the Revised International Prognostic Scoring System (IPSS-R), with adverse prognosis conferred by select mutations (i.e., *ASXL1*, *EZH2*, *TP53*). The recent introduction of the Molecular International Prognostic Scoring System (IPSS-M) for MDS [10] is a major step forward toward personalized care. Yet, established prognostic scoring systems are designed to be most informative and accurate when applied at diagnosis and prior to the initiation of therapy. Considering the numberof patients with MDS who receive immunomodulatory or hypomethylating agent therapy, the need for a more dynamic prognostic system, applicable at any time point in the course of disease and irrespective of therapy, is an unmet need. Furthermore, criteria for MDS complete remission (CR) in the bone marrow depend upon morphologic evaluation that includes the presence of <5% myeloblasts in the marrow with normal trilineage maturation [11]. These criteria have inherent limitations, as they depend on sample quality and morphologists’ experience, affecting their reproducibility and predictive value. In addition, they don’t consider underlying molecular events that may not translate into obvious morphologic abnormalities and/or increased blasts.

In this article, we provide an overview for the role of co-mutational patterns, clonal size, and hierarchy in determining clinical phenotype, prognosis, and response to therapy in patients with MDS. In addition, we provide a brief account of current and emerging promising tools to help us better refine our diagnostic and prognostic abilities in MDS.

## 2. Clonal Heterogeneity in MDS 

Mutational catalogs in MDS represent a historical record of alterations that have accumulated during life. The heterogeneity among neoplastic cells can often be used to infer the temporal order of these events [12]. Alterations identified in every sequenced neoplastic cell can be considered to form the trunk of somatic mutations’ evolutionary tree, while subclonal mutations, present in only a subset of neoplastic cells, make up the branches [12]. A neoplastic clone emerges from a single cell that has acquired one or several somatic alterations. Additional driver events that occur in individual daughter cells generate subclones, each endowed with specific functional properties and fitness [12]. Nevertheless, this intraclonal genetic diversity may not explain the entire spectrum of functional heterogeneity among individual cells within a tumor clone. Table 1 provides a glossary of genetic terms discussed below pertaining to clonal evolution in MDS.

A plethora of bioinformatic tools have been developed to help decipher the temporal order of mutations and clonality (clonal vs subclonal) in neoplastic conditions. Additionally, orthogonal tools to dissect heterogeneity using data from single-cell sequencing methods have also been developed more recently. The prevalence of subclonal mutations in different cell populations can be used to infer the clonal hierarchy of MDS. Accumulating evidence suggests that certain drivers are more likely to be subclonal than others [12]. Such differences may reflect the importance of epistasis in cancer evolution and agree with findings that co-occurrence and mutual exclusivity relationships between driver alterations can vary extensively in different cancer types. 

Furthermore, neoplastic cells are subject to selection, and the genetic variation between these cells, influenced by endogenous and exogenous processes, provides the fuel for selection to act. Although heterogeneity is required for neoplastic clones to evolve, positive selection does not necessarily lead to heterogeneity, as pressure imposed by therapy among other epigenetic factors can follow the laws of neutral growth [13]. The relationship between the number of subclonal mutations and their frequency could be consistent with a neutral growth pattern rather than subclonal expansions, leading to heterogeneity detected in MDS. Thus, both selection and neutral growth may cooperate. However, this dynamic may change over the course of time, and diversity may lead to a selection of aggressive subclones, irrespective of therapeutic pressures.

Enrichment of subclonal mutations in MDS suggests that positive selection for certain mutations is present throughout its evolutionary timeline, and that the prediction of distinct clinical behaviors depending on the presence of certain mutations at a certain point is subject to ongoing investigation, even though the occurrence of neutral evolution and drift may limit the ability to predict patterns of growth. Clonal heterogeneity and mutational diversity as assessed by whole exome sequencing (WES) seem to be lowest in low-risk MDS and increased in myelodysplastic/myeloproliferative neoplasms (MDS/MPN), high-risk MDS, and secondary AML (sAML), where it tends to be highest [9]. The limitations of using WES to decipher disease-specific genomic alterations should be noted, as the exome comprises only ~1.2% of the whole genome, and the vast majority of single nucleotide pleomorphism (~94%) occurs within non-coding genomic regions, limiting our understanding of phenotypic manifestations and therapeutic responses to treatment.

## 3. Recurrent Cytogenetic and Molecular Alterations in MDS

**Figure 1:**
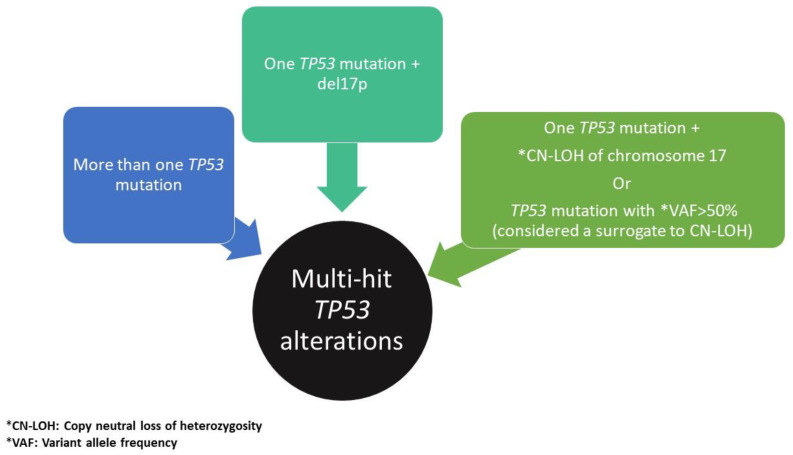
Multi-hit *TP53* defining alterations.

Copy number alterations (CNAs), defined as gains or losses of chromosome material, comprise the majority of unbalanced chromosomal aberrations in MDS [14], with s del(7q) and del(5q), followed by trisomy 8, dup(1q), del(20q), del(11q), del(12p)/t(12p), del(17p)/ iso(17q) and del(13q) representing the most common forms.: [15]. Complex karyotypes (CKs), defined as harboring >3 chromosomal aberrations, are frequently associated with *TP53* alterations(s), conferring a dismal clinical outcome [16,17,18]. Furthermore, it has been shown in a large cohort of MDS patients that the allelic state of *TP53* (mono-allelic versus bi-allelic alterations) is critical for prognostication in MDS, as multi-hit (bi-allelic) *TP53* lesions, which are found in approximately 2/3 of MDS with *TP53* aberrations, may predict risk of death and leukemic transformation independently of the IPSS-R, whereas MDS with monoallelic *TP53* alterations seems to behave similarly to MDS with wild-type *TP53* with respect to hematologic parameters and outcomes [19]. Accordingly, both the WHO 5th edition [1] and the International Consensus Classification (ICC) [20] have introduced specific MDS subclassifications accounting for cases with bi-allelic loss of *TP53* (further discussed below) (Figure 1).

A large proportion of MDS patients harbor one or more recurrent mutation(s) in association with CNAs [8,21]. Involved genetic pathways include epigenetic regulation via DNA methylation (*TET2*, *DNMT3A*, and *IDH1/IDH2*) [22,23,24], chromatin/histone modification (*KMT2D*, *EZH2*, *ARID2* and *ASXL1*) [25,26], and RNA splicing (*SF3B1*, *SRSF2*, *U2AF1*, *U2AF2*, and *ZRSR2*) [21,27,28]. Several other molecules and pathways may be involved, including *TP53* and the DNA repair machinery (PPM1D, *BRCC3*, *FANCL*, and *ATM*) [8,21], cohesion complex and associated proteins (*STAG2*, *RAD21*, *SMC1A*, *SMC3*, *NIPBL*, *PDS5B*, and *CTCF*) [29], transcription factors (*RUNX1*, *ETV6*, *GATA2*, and *IRF1*), the RAS pathway (*NRAS*, *KRAS*, *PTPN11*, *NF1*, and *CBL*) [30], and other signaling molecules (*JAK2*, *KIT*, *MPL*, *GNB1*, and less commonly *FLT3*) [8,21]. Table 2 summarizes key genetic aberrations in MDS. 

## 4. Events Influencing Mutational Order

Many mechanisms may influence the mutational hierarchy: An initial mutation alters the cellular composition of the neoplastic clone, including early stem cells and progenitors and their differentiated progeny [31]. The subsequent acquisition of a second mutation forces the double-mutantclone to reside in a cellular environment that is determined by the identity of the founder clone. Cellular interactions between genetically distinct subclones include those dictated by direct competition for available niches, as well as those imparted by feedback of differentiated cells on stem and progenitor cells [32].

As an example, Ortmann et al. have suggested that an initial (founder or ancestral) mutation may create distinct cellular pathways facilitating the acquisition of subsequent mutations that are in turn able to provide a growth advantage to the tumor. For example, the order in which the two driver events in *JAK2* and *TET2* are acquired in myeloproliferative neoplasms affects the clinical course of the disease [31]. If a *TET2* mutation is acquired first, expansion of hematopoietic stem and progenitor cells occurs, which blocks the expansion of erythroid progenitors until the cells acquire a *JAK2* mutation. Conversely, if a *JAK2* mutation is acquired first, megakaryocytic or erythroid hyperplasia becomes a dominant feature without an expansion of the hematopoietic stem cell and progenitor pool until a *TET2* mutation is acquired. Thus, patients acquiring a *JAK2* mutation first are more likely to manifest with polycythemia vera and develop thrombosis than they are to develop essential thrombocythemia [31]. 

In the setting of MDS, the founder or ancestral mutation may modify the epigenetic program of hematopoietic stem and progenitor cells and dictate the consequences of the second mutation. Mossner et al. [33] showed that mutations affecting epigenetic modifiers (e.g., *TET2* and *ASXL1*) and RNA splicing factors (e.g., *SF3B1* and *SRSF2*) are predominantly “founder” events in MDS [33]. Genes involved in signaling cascades (e.g., *JAK2* and *CBL*), transcription factors (e.g., *RUNX1* and *ETV6*), and chromosomal alterations are almost exclusively acquired as late events in MDS. This contrasts with reports proposing del(5q) as the initiating lesion in isolated del(5q) patients [34]. This emphasizes the potential use of mutational order as an indicator of disease progression. Nevertheless, a typically labeled “founder” lesion may appear as a secondary hit in another patient, highlighting the role of “patient-specific” molecular characterization in predicting disease phenotype and course and guiding therapeutic decision.

Nakashima et al. [9] identified two main classes of mutations in MDS. Type 1 mutations included those involving *FLT3*, *PTPN11*, *WT1*, *IDH1*, *NPM1*, *IDH2*, and *NRAS* and were enriched in sAML in comparison to *de novo* high-risk MDS. Type 2 mutations involved *TP53*, *GATA2*, *KRAS*, *RUNX1*, *STAG2*, *ASXL1*, *ZRSR2,* and *TET2* and were enriched in high-risk MDS, in comparison to low-risk MDS. The authors also found that type-1 mutations in sAML were more likely to be acquired at the time of transformation, whereas type-2 mutations were more frequently persistent throughout the course of the disease [9]. In addition, the clinical course of patients with type-1 mutations was characterized by a shorter time to progression to AML [9]. On the other hand, a low incidence of progression to AML was observed in patients harboring *SF3B1* mutations, without type-1 or type-2 mutations [9]. These mutational classes were demonstrated to be independent predictors of progression-free survival (PFS) in multivariable analysis [9]. The impact of these mutations on disease biology highlights their potential utility in disease monitoring [9].

Nevertheless, in this study there were some overlaps between these mutation categories, suggesting that the biological impact of mutations may also be impacted by the dynamics of mutation acquisition and clonal hierarchy and not simply by the affected pathways.

## 5. Clonal-Rank vs. Burden: Hierarchy Matters 

Nakashima et al. [9] showed that patients with MDS with larger clones with type-1 mutations, suffered from shorter progression-free survival (PFS) in comparison to those that had smaller clones with type 1 mutations. However, overall survival (OS) was not affected by clonal size (burden) [9]. On the other hand, among patients with MDS with larger clones with type-2 mutations, OS was shorter, however, clonal size did not affect PFS [9].

While variant allelic frequency (VAF)-based clonal ranking (i.e., clonal size and/or burden) has been used to infer clonal hierarchy in myeloid neoplasms, the methodological feasibility of VAF-based clonal ranking is limited at best. Using a similar methodology, Awada et al. [35] found that the specific disease phenotypes and clinical outcomes of patients with myeloid neoplasms with *SF3B1* mutations are dictated by the clonal size and hierarchy of these mutations.

In addition, several studies have highlighted that specific mutation types (canonical versus non-canonical; missense vs truncating, etc.) seem to affect hierarchical rank as well. For example, *DNMT3A* R882 mutations tend to be dominant in comparison to other mutations (i.e., truncating, other missense mutations etc.), which tend to be secondary events [36].

## 6. Ancestral Versus Secondary, Random Versus Pre-Determined Clones

Clonal hierarchy affects the role of individual mutations, with an impactful presence asserted by ancestral clones versus secondary or subclonal mutations. By analyzing 1809 MDS patients, Nagata et al. inferred the clonal architecture using a stringent single-cell sequencing platform that deciphered the position of various mutations [36]. The authors grouped mutations into ancestral versus secondary, as inferred by their rank in the clonal hierarchy, and correlated their findings with disease morphology, progression, survival, and response to therapy. They found that early (ancestral) mutations often involved *SF3B1* and *U2AF1*, while mutations in *ASXL1*, *CBL*, and *KRAS* were more likely to be late (secondary) mutations and associated with an increased risk of disease progression, irrespective of the clone size [36]. 

Several elegant genomic studies have elucidated the presence of functional interactions between somatic mutations in MDS, reinforcing the concept of positive and negative selections. These studies have shown that secondary genetic hits in MDS are not random and follow a certain order. This fundamental observation is supported by the fact that certain mutational combinations occur more commonly in MDS [36], while others are mutually exclusive.

Individual mutations may impact clonal hierarchy depending on their position (ancestral versus secondary events, irrespective of the nominal clonal burden [36]. Ref. [37] Significant relationships among distinct combinations of dominant and/or secondary mutations have been identified, with subsets affecting phenotypes, prognosis, and response to hypomethylating agents (HMAs) [36], as outlined in the following section. 

## 7. Impact of Mutation Combination on Phenotype and Response to Therapy

The differential impact of mutational combinations on disease phenotype (MDS, MDS/MPN), prognosis (low- or high-risk), and response to HMAs has been elucidated [36]. For example, the acquisition of an *SRSF2* mutation in a clone harboring a pre-existing *TET2* mutation often results in a tendency to develop a high risk MDS/MPN disease, often with monocytosis [36]. Similarly, co-existence of *EZH2* as a dominant clone with *ASXL1* or *RUNX1* mutations as secondary clones correlates with a high-risk disease and poor prognosis disease [36].

To study the effect of mutational combination on disease phenotype, Nagata et al. [36] ranked combinations based on the odds of MDS versus MDS/MPN association or low-risk versus high-risk disease. For instance, del(5q), *STAG2* mutations, and complex karyotype were more likely to be associated with MDS features, while *JAK2*, *EZH2*, and RAS pathway (*NRAS/KRAS/CBL*) mutations were associated with MDS/MPN overlap features. Similarly, *RUNX1*, *STAG2* mutations, and -7/del(7q) were more likely to group with high-risk subtypes, in contrast to *SF3B1* and *JAK2*, which constituted mutations predictive of low-risk subtypes.

Distinction of dominant hits may also be predictive of response to therapy. It has been recognized that the presence of *TET2* mutations is predictive of responsiveness to HMAs, while *ASXL1* mutations are predictive of refractoriness to HMA therapy [36]. Nevertheless, while MDS harboring dominant *TET2* mutant clones seem to have a better response to HMA therapy, MDS with secondary and/or subclonal *TET2* mutations tends to exhibit a weaker treatment response. In contrast, patients with MDS harboring secondary *ASXL1* mutations tend to be statistically less likely to obtain CR [36]. Overall, patients with MDS harboring *TET2* mutations and wild type *ASXL1* seem to be the most likely group to show the best response to HMA therapy.

## 8. Updates to MDS Classification Incorporating Additional Genetic Factors: WHO vs. ICC

Both the WHO 5th edition classification [1] and the ICC [20] now classify MDS in two overarching categories: (a) MDS with defining genetic abnormalities; and (b) morphologically defined MDS. The former category in both classifications includes MDS with isolated del 5q, MDS with *SF3B1* mutation, MDS with bi-allelic *TP53* inactivation (WHO) (Figure 1), and MDS with mutated *TP53* (ICC). There are subtle differences in how the two systems designate MDS with *SF3B1* mutation, in that the WHO also recognizes cases with increased (≥15%) ring sideroblasts without *SF3B1* mutation as a continuum of the *SF3B1* mutated category, allowing for the use of MDS with RS as an alternative designation in order to account for cases with similar biology driven by similar genetic lesions such as those involving other splicing factors, whereas the ICC classifies such cases as MDS, not otherwise specified. In addition, the WHO uses a blast threshold of 20% to define AML transformation from MDS in the category of MDS with biallelic *TP53* inactivation (albeit with a note that MDS with increased blast-2 (≥10% blasts) may be regarded as “AML-equivalent” for therapy and clinical trials enrollment, when appropriate). In contrast, the ICC introduces a novel category of MDS/AML for cases with *TP53* mutation (regardless of allelic state) with blasts ≥10%. While the expansion of the overarching category of MDS with defining genetic alterations in both systems is a welcome change, additional validation studies are needed to rectify the subtle differences that currently exist between these two systems.

## 9. Prognostic Scoring Systems: From IPSS to IPSS-R and IPSS-M

The International Prognostic Scoring System (IPSS) for primary MDS was introduced in 1997 by Greenberg et al. [38]. Elements of the IPSS are bone marrow blast percentage (<5% vs. 5–10% vs. 11–20% vs. 21–29%), karyotype (good, intermediate or poor), and number of cytopenias (0–1 vs. 2–3), where cytopenia(s) are defined by hemoglobin <10 g/dL, platelets <100 × 10^9^/L, and leukocyte count <1.8 × 10^9^/L). Cytogenetic groups in IPSS include the following: good (diploid karyotype, -Y, del(20q), del(5q), poor (-7/del(7q) or complex karyotype), and intermediate (all other abnormalities). The simplicity and efficacy of IPSS in readily isolating MDS patients who might benefit from aggressive therapy, such as stem cell transplant, made it very appealing in clinical settings.

IPSS was revised in 2012, and a more sophisticated version, IPSS-R, was created [39]. IPSS-R components were further edited as follows: Bone marrow blast percentage (≤2% vs. >2 and <5% vs. 5–10% vs. >10%), hemoglobin level (<8 vs. >8 and <10 vs. 10 g/dL), platelet count (<50 vs. >50 and <100 vs. 100 × 10^9^/L), and absolute neutrophil count (0.8 vs. <0.8 × 10^9^/L. IPSS-R suggested five cytogenetic groups: (1) very good (-Y, del(11q); (2) good (normal, del(5q), del(20q), del(12p) or two abnormalities including del(5q); (3) intermediate (+8, del(7q)-, i(17q), +19, +21, other single abnormalities, independent clones, two abnormalities not including del(5q) or -7/del(7q); (4) poor (-7, inv(3), del(3q), two abnormalities including del(7q), complex karyotype with 3 abnormalities); (5) and very poor (complex karyotype with >3 abnormalities) [40].

The value of IPSS-R in MDS is hindered by its complexity, inconsistent reproducibility, and limited application in previously untreated MDS patients. Subsequent reviews have been published to address some of these limitations [41,42,43]. Furthermore, IPSS-R considered hematologic parameters and cytogenetic abnormalities only, without taking into account somatic gene mutations to assess the risk stratification of patients with MDS. 

Recently, a clinical-molecular prognostic model (IPSS-Molecular [IPSS-M]) [10] was developed and validated. Profiling 152 genes, the investigators identified multi-hit *TP53* alterations, *FLT3* mutations, and *KMT2A*^PTD^ as top genetic predictors of adverse outcomes. Furthermore, depending on patterns of co-mutations, *SF3B1* mutations were shown to be associated with favorable outcomes. This scoring system uses hematologic parameters (including blood counts and bone marrow blasts), cytogenetic abnormalities, and somatic mutations of 31 genes to stratify MDS patients into five risk categories: very low, low, moderate, high, and very high. Applying this model to MDS patients previously classified using IPSS-R led to re-stratification of 46% of patients, of which 74% were up-staged and 26% were down-staged. Furthermore, an IPSS-M Web calculator (https://mds-risk-model.com, accessed on 12 June 2022) has been developed to help integrate this scoring system seamlessly into clinical practice. A comparative evolution of parameters incorporated in the three above-mentioned scoring systems is illustrated in Table 3. 

Although IPSS-M is a positive step in the right direction, and has proven to improve prognostic discrimination across all clinical end points, the applicability of this system is restricted to primary- and therapy-related MDS only, prior to the initiation of therapy. This highlights the need for a more dynamic system that would be applicable at any time point of disease evolution, irrespective of therapy. Furthermore, the IPSS-M demonstrated that the outcome of *SFRB1* mutations in particular (previously recognized as generally “favorable”) depends on the *SF3B1* mutational subtype and its combinational patterns with co-existing mutations in other genes. This finding highlights the importance of an in-depth investigation of clonal hierarchy in MDS using single-cell sequencing, to tease out mutational combinations with prognostic significance in future re-iterations of the scoring system.

## 10. Clonal Hematopoiesis Ancestral Relationship to MDS

Clonal hematopoiesis (CH) with indeterminate potential (CHIP) or CH with oncogenic potential (CHOP) is defined by the presence of hematopoietic cells carrying somatic mutations and CNAs overlapping with MDS/AML with a VAF>2% and without cytopenias or morphologic dysplasia. These include *DNMT3A*, *TET2*, *ASXL1*, *SF3B1*, *SRSF2*, *CBL*, *U2AF1*, and *IDH1-2*, as well as del(20q), del(13q), 14qUPD, and del(11q)/11qUPD [44,45,46]. The occurrence of CH yields a ten-fold (for mutations [47,48]) to thirty-fold (for CNAs [44,45]) higher risk of evolution to a myeloid neoplasms, with higher risks observed in cytopenic patients with CH; i.e., otherwise known as clonal cytopenia of undetermined significance (CCUS) [1,49]. 

**Figure 2:**
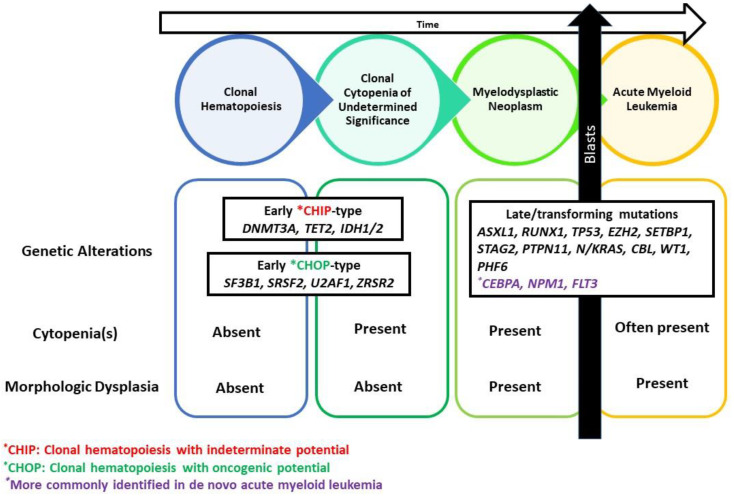
Mutations contributing to myelodysplastic neoplasms (MDS) disease establishment and progression. Clonal hematopoiesis of indeterminate potential or oncogenic potential harboring ancestral and/or founder mutations in a specific set of genes is characterized by the absence of cytopenia(s) and morphologic dysplasia. Clonal hematopoiesis with acquired persistent cytopenia(s) is defined as CH in the presence of cytopenias but without morphologic evidence of dysplasia. Evidence of morphologic dysplasia in the presence of clonality and cytopenias is diagnostic of MDS. Acquisition of late and/or transforming mutations leads to evolution into acute myeloid leukemia.

The striking overlap of early and/or founder events identified in MDS and other myeloid neoplasia [50] is suggestive of a common CHIP/CHOP-like phase in MDS followed by more confined diversification through the acquisition of late and/or transforming aberrancies [33,51], indicating that CH-related genetic lesions are ancestral events to MDS (Figure 2). Nevertheless, the risk of evolution into MDS or AML remains minimal in general (~1% per year on average) and seems to correlate with clonal burden and architecture [52]. Mutations in *TP53* and spliceosome factors, particularly *U2AF1* and *SRSF2* correlate with a higher risk of transformation into a bona fide myeloid neoplasms [53]. *DNMT3A* and *TET2* mutations on the other hand are most common in CH but are associated with a lower risk of evolution into myeloid neoplasms [52,54]. (Table 2).

It is speculated that a subset of CHIP plays a role as an ancestral hit, with acquisition of additional genetic alterations and evolution into MDS. This natural history of MDS arising from antecedent CHIP may be distinct from MDS harboring CHOP mutations [36,51]. Dominant mutations in *DNMT3A, TET2*, and *ASXL1*, which occur more frequently in CHIP than in MDS, have been characterized as early “CHIP-type” MDS mutations [36,51]. Conversely, dominant mutations in *U2AF1, RUNX1*, and *STAG2* are characterized as early “CHOP-type” MDS mutations. (Figure 2) [36,51].

## 11. Transcriptomic Data & the Road to Improving MDS Stratification

Many transcriptomic-based scoring systems have been suggested to improve the current risk stratification of MDS patients. For example, using transcriptomic data of CD34+ cells from 159 MDS patients and 17 healthy donors, Coelho-Silva et al. [55] selected 37 genes involved in cellular energetics and interrogated their clinical and prognostic functions. Based on the low expression of ACLY, ANPEP, and PANK1, as well as the high expression of PKM and SLC25A5, they constructed a molecular-based score that categorized patients in the following three risk groups: favorable risk, intermediate, and adverse. Transcriptional signature revealed that favorable- and intermediate-risk patients presented enriched molecular programs related to mature myeloid progenitors, cell cycle progression, and oxidative phosphorylation, indicating that these cells differ in their origin, metabolic state, and cell cycle regulation, in comparison to the adverse-risk group. Their study provided evidence that cellular energetics is transcriptionally deregulated in MDS CD34+ cells and establishes a new useful prognostic score based on the expression of five genes.

Thol et al. [56] combined the expression data of *MN1*, *ERG*, *BAALC*, and *EVI1* (MEBE) (associated with poor prognosis in patients with cytogenetically normal AML) to study their prognostic effects in MDS. The authors investigated the expression data of these four genes from 140 MDS patients in an additive score, which was validated in an independent patient cohort of 110 MDS patients. A high MEBE score, defined as the high expression of at least two of the four genes, predicted a significantly shorter overall survival (OS) and time to AML progression, compared to a low MEBE score in multivariate analysis independent of karyotype, percentage of bone marrow blasts, transfusion dependence, *ASXL1*, and *IDH1* mutational status. In a validation cohort of 110 MDS patients, a high MEBE score predicted shorter OS and time to AML progression. A high MEBE expression score is an unfavorable prognostic marker in MDS and is associated with an increased risk for progression to AML. Expression of the MEBE genes is regulated by *FLI1* and c-*MYC*, which are potential upstream targets of the MEBE signature.

Yao et al. [57] profiled long noncoding RNAs (lncRNAs) expression (known to participate in normal hematopoiesis but also contribute to the pathogenesis of acute leukemia) in 176 adult patients with primary MDS and identified four lncRNAs whose expression levels were significantly associated with overall survival (OS). They then constructed a risk-scoring system with the weighted sum of these four lncRNAs. Higher lncRNA scores were associated with higher marrow blast percentages, higher-risk subtypes of MDSs (based on both IPSS-R and world health organization classification), complex cytogenetic changes, and mutations in *RUNX1*, *ASXL1*, *TP53*, *SRSF2*, and *ZRSR2*, whereas they inversely correlated with *SF3B1* mutation. Patients with higher lncRNA scores had a significantly shorter OS and a higher five-year leukemic transformation rate compared with those with lower scores. In multivariate analysis, higher lncRNA scores remained an independent unfavorable risk factor for OS irrespective of age, cytogenetics, IPSS-R, and gene mutations. The integrated 4-lncRNA risk-scoring system correlated with distinctive clinical and biological features in MDS patients and served as an independent prognostic factor for survival and leukemic transformation. 

## 12. The Role of Flow Cytometry Analysis in MDS Follow-Up

Establishing a diagnosis of MDS entails a comprehensive approach with a focus on the morphological abnormalities of the various hematopoietic cell lineages as well as evaluation for evidence of clonality. The current diagnostic criteria for MDS require the presence of morphologic dysplasia to distinguish cases of MDS from clonal cytopenia of undetermined significance (CCUS). However, the assessment of morphologic dysplasia can be subjective, particularly when these changes are mild, for example in cases of low-grade MDS, and evaluation may be limited by the quality of bone marrow biopsy and aspirate specimens in cases with suboptimal sampling. On the other hand, morphologic dysplasia is not specific and may be a manifestation of non-neoplastic conditions. Therefore, it is critical to utilize all tools in our armamentarium when a diagnosis of MDS is in consideration. One particular useful tool is flow cytometry immunophenotyping (FCI).

Although FCI is not currently incorporated as a diagnostic criterion or means of follow-up for MDS in the WHO Classification scheme [1], increasing evidence supports the addition of FCI as a tool for the diagnostic evaluation of MDS [58,59,60]. In fact, the application of FCI in the diagnostic work-up has been the subject of extensive studies, and different FCI-based scoring systems have been developed for this indication. These include the “Ogata score” [59,61], the “Wells algorithm” [62,63], and the integrated flow cytometry (iFC) score developed by the international consortium and the European LeukemiaNet (ELN) Working Group [64,65]. The latter has proposed minimal requirements for the standardization of FCI in the diagnostic work-up of MDS [65]. These include: (1) Quantification of CD34+ myeloid progenitors; (2) Quantification of B-cell progenitors or hematogones as a fraction of the CD34+ cell population; (3) Assessment of aberrant or altered antigen expression on myeloid progenitor cells and maturing myelomonocytic cells; and (4) Assessment of cytoplasmic granularity of maturing granulocytes using their side-scatter property. Among these groups, the most reliable cellular compartment for assessment in cases of suspected MDS seems to be the CD34+ myeloid blast population, as the maturing myeloid and monocytic cells may show immunophenotypic alterations in cases of reactive cytopenia or bone marrow regeneration. Currently, criteria for MDS’s CR in the bone marrow depend upon morphologic evaluation that includes the presence of <5% myeloblasts in the marrow with normal trilineage maturation [11]. These criteria depend on the interpreters experience and may not be completely reproducible. FCI in this particular setting represents a potentially attractive tool for with the surveillance of patient with MDS. Studies to evaluate the normalization or improvement of hematopoietic stem cell phenotypes in the setting of HMA therapy would provide useful information in this regard. 

## 13. Concluding Remarks

In summary, based on current available literature, clonal diversity, dynamics, and associated events play key roles in MDS outcomes and modes of progression. The presence of several coexisting mutations alters the prognosis of MDS patients. Throughout clonal evolution, the phenotypic and biologic features of MDS are remodeled by several driver mutations and a predilection for a specific spectrum of subsequent secondary mutations. Our ability to identify individual mutational hierarchies is a necessary step towards personalized therapy. Such knowledge could guide patient-specific drug targeting of particular clones with the aim of eradicating MDS by targeting the “founder” lesion, or eradicating advanced clones, allowing for hematopoietic reconstitution and hematologic improvement. The newly available IPSS-M model identified *TP53* multi-hit mutations, *FLT3* mutations, and *KMT2A*^PTD^ as top genetic predictors of adverse outcomes and set the groundwork for future classifications system that would ideally take into consideration all genomic aberrations and their inferred clonal architecture and hierarchy in individual cases. Moving forward, correlation of mutational data with these and other ‘-omic’ data sets may further refine our understanding of MDS biology, improving outcome prediction and treatment choice. Meanwhile, deploying FCI to assess responses in MDS cases is a potential option in our repertoire. Although its integration is not standardized yet, it nevertheless continues to add valuable information that may be otherwise missed by conventional morphologic examinations of post-treatment MDS marrows.

## Figures and Tables

**Table 1:** Glossary of major concepts pertaining to clonal evolution in myelodysplastic neoplasms.

Term	Definition
Clonal Hierarchy	The order in which mutations are acquired. “Founder” (ancestral) mutations, typically involving epigenetic modifiers and splicing factors in MDS, occur early and are followed by “successor” alterations, which typically involve the signaling cascades, transcription factors, and copy number alterations
Clonal Burden	Size of a population with a specific genetic aberrancy (for mutations, typically inferred using variant allele frequency “VAF” by next-generation sequencing analysis)
Clonal Sweeping	The process by which a neoplastic population develops new advantageous genetic lesions, allowing it to expand and dominate the neoplasm (“selective sweeps” or “positive selection”)
Neutral Growth	Egalitarian propagation of neoplastic populations harboring genetic aberrations that do not affect cellular fitness
Clonal Heterogeneity	Genetic diversity among neoplastic populations resulting from an interplay between clonal sweeping and/or neutral growth
Mutational Combination	Pre-determined (in contrast to random) co-occurrence of certain mutations resulting in a specific disease phenotype
Epistasis	The conditional relationship between two genes that can determine a single phenotype (an allele of one gene hides or masks the phenotype of another gene)

**Table 2:** Key genetic landscape in myelodysplastic neoplasms (MDS). Recurrent abnormalities can be broadly divided into mutations and cytogenetic aberrations. The most common cytogenetic alterations include del(7q), del(5q) & trisomy 8. Recurrent mutations in MDS affect several cellular pathways and functions, most commonly DNA methylation, chromatin/histone modification, RNA splicing, cohesion complex, the RAS pathway, DNA repair machinery, and several signaling molecules. Recurrent mutations can be further divided into early “CHIP (clonal hematopoiesis with indeterminate potential)-type” affecting genes commonly involved in clonal hematopoiesis, “CHOP (clonal hematopoiesis with oncogenic potential) -type,” and late and/or transforming events that affect genes not classically associated with clonal hematopoiesis. Three genetic events are MDS class-defining according to the 5th edition of the World Health Organization (WHO) classification and the International Consensus Classification (ICC), these include isolated del(5q), *SF3B1* mutations and multi-hit/bi-allelic *TP53* alterations. The International Prognostic Scoring System-Molecular (IPSS-M) identifies multi-hit *TP53* alterations, *FLT3* mutations, and *KMT2A* partial tandem duplication as the top three genetic predictors of leukemic transformation and adverse outcomes in patients with MDS.

Recurrent Cytogenetic Abnormalities
**Most common:** del(7q), del(5q) & + 8
**Others:** dup(1q), del(20q), del(11q), del(12p)/t(12p), del(17p)/ i(17q), del(18q), 12q gains, del(13q), der(1;7)(q10;p10)
**Recurrent Mutations**
**DNA methylation:***TET2*, *DNMT3A* & *IDH1/IDH2*
**Chromatin/histone modification:***KMT2D, EZH2, ARID2* & *ASXL1*
**RNA splicing:***SF3B1*, *SRSF2*, *U2AF1, U2AF2* & *ZXRSR2*
**Cohesin complex:***STAG2*, *RAD21*, *SMC1A*, *SMC3*, *NIPBL*, *PDS5b* & *CTCF*
**Transcription:***RUNX1*, *ETV6*, *GATA2* & *IRF1*
**RAS pathway:***NRAS*, *KRAS*, *PTPN11*, *NF1* & *CBL*
**DNA repair machinery:***TP53*, *PPM1D*, *BRCC3*, *FANCL* & *ATM*
**Signaling molecules:***JAK2*, *KIT*, *MPL*, *GNB1* & *FLT3*
**Early CHIP (clonal hematopoiesis with indeterminate potential)-type**
*DNMT3A*, *TET2*, *ASXL1*, *IDH2*
**CHOP (clonal hematopoiesis with oncogenic potential)-type**
*U2AF1*, *SRSF2*, *ZRSR2*, *SF3B1*
**Late/ Transforming mutations**
*ASXL1*, *RUNX1*, *TP53*, *EZH2*, *SETBP1*, *STAG2*, *NPM1*, *FLT3*, *PTPN11*, *N/KRAS*, *CBL*, *WT1*, *PHF6*
**MDS-Defining Genetic Abnormalities Per WHO & ICC**
Isolated del(5q), *SF3B1*, Multi-hit/bi-allelic *TP53* mutations
**IPSS-M Top Predictors of Adverse Outcomes**
Multi-hit *TP53* alterations, *FLT3* mutation, *KMT2A* partial tandem duplication

**Table 3:** Evolution of Prognostic Scoring Systems in Myelodysplastic Neoplasms.

Evolution of Prognostic Scoring Systems in Myelodysplastic Neoplasms				
**Variables in the International Prognostic Scoring System (IPSS)-1997**				
**Blast Percentage** <5% 5–10% 11–20% 21–29%	**Cytopenia(s)** -Hemoglobin <10 g/dL -Platelets <100 × 10^9^/L -Leukocyte count <1.8 × 10^9^/L)		**Cytogenetic Groups** -Good (diploid karyotype, -Y, del(20q), del(5q) -Poor (chromosome 7 anomalies or complex karyotype with 3 abnormalities) -Intermediate (all other abnormalities)	
**Variables in the Revised International Prognostic Scoring System (IPSS-R)-2012**				
**Blast Percentage** ≤2% >2 and <5% 5–10% >10% and <20%	**Cytopenia(s)** -Hemoglobin <8 vs. >8 and <10 vs. 10 g/dL -Platelet count <50 vs. >50 and <100 vs. 100 × 10^9^/L -Absolute neutrophil count 0.8 vs. <0.8 × 10^9^/L		**Cytogenetic Categories** -Very good (-Y, del(11q) -Good (normal, del(20q), del(5q)del(12p)- or two abnormalities including del(5q) -Intermediate (+8, del(7q), i(17q), +19, +21, other single abnormalities, independent clones, two abnormalities not including del( 5q) or -7/del(7q) -Poor (-7, inv(3), del(3q), two abnormalities including del(7q), complex karyotype with 3 abnormalities) -Very poor (complex karyotype with >3abnormalities)	
**Variables in the Molecular International Prognostic Scoring System (IPSS-M)-2022**				
**Blast Percentage**	**Hemoglobin level**	**Platelet Count**	**IPSS-R Cytogenetic Category**	**Top Molecular Predictors of Adverse Outcome among 31 genes** -Multi-hit *TP53* alterations *-FLT3* mutation(s) *-KMT2A* partial tandem duplication
